# Comparative effective dose of ciprofol and propofol in suppressing cardiovascular responses to tracheal intubation

**DOI:** 10.1038/s41598-025-85968-2

**Published:** 2025-01-13

**Authors:** Min Liao, Xiao-Ru Wu, Jia-Ning Hu, Xing-Zhou Lin, Tang‑yuan‑meng Zhao, Hu Sun

**Affiliations:** 1 The Second Affiliated Hospital of Hainan Medical University, Hai Kou, China; 2 West China Hospital, Sichuan University, Chengdu, 610041 China

**Keywords:** Propofol, Ciprofol, Tracheal intubation, Cardiovascular response, Dose-response relationship, Drug discovery, Anatomy, Diseases, Medical research

## Abstract

Ciprofol, a novel γ-aminobutyric acid receptor agonist, outperforms propofol with minimal cardiovascular effects, higher potency, reduced injection pain, and a broader safety margin. Despite these advantages, ciprofol’s clinical research is still emerging. This study compares the median effective dose (ED_50_) and adverse reactions of ciprofol and propofol, in conjunction with sufentanil, for suppressing cardiovascular responses during tracheal intubation. Fifty-three adult patients scheduled for tracheal intubation under general anesthesia were enrolled and randomly assigned to receive either ciprofol (Group C) or propofol (Group P), according to a random number table. Tracheal intubation was performed using a standardized laryngoscope and endotracheal tube. The Dixon’s up-and-down method was employed to determine the ED_50_ and 95% effective dose (ED_95_) of ciprofol and propofol in inhibiting cardiovascular responses during tracheal intubation. Based on the pilot study, the initial dose for ciprofol was set at 0.35 mg/kg (with a 0.01 mg/kg increment) and for propofol at 2.0 mg/kg (with a 0.1 mg/kg increment). Probit analysis was applied to derive dose-response curves, while adverse reactions were continuously monitored. A total of 54 participants were included, with 24 in group C (1 excluded) and 30 in group P. Probit analysis revealed that the ED_50_ of ciprofol for inhibiting cardiovascular responses to tracheal intubation were 0.326 mg/kg (95% CI 0.304–0.337 mg/kg), and for propofol, 1.541 mg/kg (95% CI 1.481–1.599 mg/kg). The heart rate in group P was significantly higher than the group C at 1 minute (*p* = 0.026) and 3 minutes (*p* = 0.016) post-intubation. Systolic and diastolic blood pressures (SBP and DBP) decreased significantly before and after intubation compared to baseline values in both groups (*p*< 0.05). Group C experienced significantly less injection pain (*p* = 0.001), although the incidence of other adverse effects was not statistically different between groups (*p* > 0.05).

*Clinical Trial Registration*: hppts://ClinicalTrials.gov; Identifier: NCT06095570(18/10/2023).

## Introduction

Laryngoscope insertion and tracheal intubation during anesthesia induction often cause patient discomfort and significant nociceptive stimulation. These procedures typically induce marked hemodynamic responses, including tachycardia, hypertension, and increased intracranial pressure^1^. These responses are caused by the stimulation of oropharyngeal and peripharyngeal tissues during laryngoscope insertion and tracheal tube placement, which activates the sympathetic nervous system and triggers increased catecholamine secretion^2^. These processes can result in dangerous circulatory instability, which is particularly risky for patients with cardiovascular or cerebrovascular conditions, and may lead to severe complications such as cerebral hemorrhage or cardiac failure^3^.

Research indicates that the combined administration of sufentanil and propofol effectively mitigates adverse cardiovascular reactions during endotracheal intubation^4^. Furthermore, multiple clinical studies have demonstrated the effective attenuation of laryngeal reflexes and reduction in the occurrence of coughing or laryngospasm after tracheal intubation through the use of propofol as a general anesthetic. Thus, propofol facilitates smoother tracheal intubation during anesthesia induction^5^. Despite its advantages, propofol has several limitations, including injection pain, dose-dependent respiratory depression, hypotension, and propofol infusion syndrome^6,7^. These issues compromise the quality of anesthesia and limit its broader clinical application.

Ciprofol, a novel phenol derivative ether, enhances GABA-mediated chloride ion influx, offering sedative or anesthetic effects^8^. Early studies highlight ciprofol’s advantages over propofol, including minimal respiratory and circulatory impact, higher potency, reduced injection pain, and a broader safety margin^8,9^. However, limited research exists on ciprofol’s optimal dosage for suppressing cardiovascular responses during tracheal intubation. This study aims to compare the ED_50_ and safety profiles of ciprofol and propofol in inhibiting cardiovascular reactions during tracheal intubation using the up-and-down method. By determining the equipotent dosages of these drugs, the study seeks to provide valuable clinical insights for anesthesia practice.

### Ethics

The Ethics Committee of the Second Affiliated Hospital of Hainan Medical University approved this study (LW202282), which has also been registered on ClinicalTrials.gov (NCT06095570, 18/10/2023). Prior to participation, all study subjects have obtained informed consent from both the patients themselves and their families, which has been documented through the signing of consent forms.

### Patient selection

Patients scheduled for elective tracheal intubation under general anesthesia at our hospital, regardless of gender, aged between 18 and 65, with a BMI ranging from 18 to 28 kg/m^2^, ASA (American Society of Anesthesiologists) class I or II, Mallampati class I or II, normal mouth opening, and normal head and neck mobility will be included. Exclusion criteria include a difficult or potentially difficult airway, uncontrolled or poorly controlled systemic diseases (hypertension, diabetes, e.g.), pregnancy, alcohol abuse, allergy to investigational drugs, history of illegal drug use, neurological disorders, or inability to effectively communicate. Additionally, failed initial tracheal intubation, emergency situations during induction, and the use of vasoactive drugs are rule-out criteria.

### Randomization and blinding

Participants meeting the inclusion criteria were randomly assigned to groups C and P using a random number table, with randomization performed by an external statistician. The anesthesia procedures involved two anesthesiologists and a nurse anesthetist. One anesthesiologist handled intubation and managed any adverse effects, while the second anesthesiologist, blinded to group assignments, recorded the observations. The nurse anesthetist administered the test drug based on the patient’s weight.

### Sample size

This study used a modified sequential design, with the sample size determined by the occurrence of 7 foldbacks, as prior research suggests that ≥ 6 foldbacks provide reliable conclusions^10^. This approach was employed to enhance the accuracy of the data at lower cost.

### Anaesthesia methods and technical routes

All patients undergo an 8-hour fasting period before surgery, without specific preoperative medications. In the pre-anesthetic room, standard intravenous access is established, and patients are randomly assigned to the Ciprofol (C) or Propofol (P) groups using a random number table. Upon entering the operating room, a reconfirmation of the patient’s identity, surgical details, and condition is conducted, while a Dash4000 monitor is employed to monitor vital signs like non-invasive blood pressure (NBP), electrocardiogram (ECG), heart rate (HR), pulse oximetry (SpO_2_), and the bispectral index (BIS).

Patients receive oxygen at 6 L/min for 3 min prior to anesthesia induction. The first patient in group C is intravenously administered Ciprofol Injection (Enterprise: Liaoning Haisi Pharmaceutical Co., Ltd, Batch number: 20220325, IV injection time > 30s) at a dose of 0.35 mg/kg, while the first patient in the P group is intravenously administered Propofol Emulsion Injection (Enterprise: Xi’an Libang Pharmaceutical Co., Ltd, Batch number: L06841, IV injection time > 30s) at a dose of 2.0 mg/kg. Once the patient loses consciousness, with the eyelash reflex gone and a Modified Observer’s Assessment of Alertness/Sedation (MOAA/S) score (Table 1) ≤ 1, Sufentanil Citrate Injection (Enterprise: Yichang Renfu Pharmaceutical Co., Ltd, Batch number: 21A11021, IV injection time: 30s) is administered at a dose of 0.25 ug/kg and Rocuronium Bromide Injection (Enterprise: Zhejiang Xianju Pharmaceutical Co., Ltd, Batch number: EA2276, IV injection time: 15s) at a dose of 0.6 mg/kg. Subsequently, under the supervision of a skilled anesthesiologist, a visual laryngoscope is used for tracheal intubation, while a stethoscope is utilized to auscultate and monitor end-tidal carbon dioxide pressure (PetCO_2_). After intubation, mechanical ventilation is initiated with a tidal volume of 6–8 ml/kg and an inspiratory/expiratory ratio of 1:2. The respiratory rate is adjusted to maintain PetCO_2_ between 35 and 45 mmHg. Anesthesia is maintained with 1% sevoflurane, ensuring BIS values remain between 40 and 60 within 3 min, without any surgical intervention. Women are intubated with a 7.0 mm endotracheal tube, and men with a 7.5 mm tube.

**Table 1:** Modified Observer’s Assessment of Alertness/Sedation (MOAA/S) scale.

Scale	MOAA/S Scale
0	Does not respond to painful trapezius squeeze
1	Responds only after painful trapezius squeeze
2	Responds only after mild prodding or shaking
3	Responds only after name is called loudly and/or repeatedly
4	Lethargic response to name spoken in normal tone
5	Responds readily to name spoken in normal tone

The study was conducted using an up-and-down method. Based on the preliminary trial, the initial dose for the ciprofol group was determined to be 0.35 mg/kg, with a dose increment of 0.01 mg/kg. For the propofol group, the initial dose was 2.0 mg/kg, with a dose increment of 0.1 mg/kg. Cardiovascular response to intubation determined dose adjustments for subsequent patients. A positive response led to a dose increment for the next patient, while a negative response resulted in a dose reduction. Our study ends after alternating 7 positive and negative responses in both groups^11^.

### Positive reaction

According to the methods mentioned in relevant literature^12^, the criteria for a positive cardiovascular response is a ≥ 20% rise in SDP/DBP or HR within 3 min post-intubation compared to baseline. A lack of such rise is considered a negative response. Additionally, if the MOAA/S score^13^ remains > 1 after 3 min of propofol or ciprofol administration, it is classified as a positive response, prompting continued administration until the score is ≤ 1.

### Outcomes

The primary outcome is the determination of the ED_50_ and ED_95_ of propofol and ciprofol in suppressing the patient’s response to tracheal intubation, calculated using haemodynamic indices such as heart rate (HR) and mean arterial pressure (MAP). Haemodynamic data were collected at the following time points: baseline (average of three measurements after admission, T1), after intravenous sedative drugs (T2), at MOAA/S ≤ 1 min (T3), immediately before intubation (T4), at intubation (T5), 1 min post-intubation (T6), 2 min post-intubation (T7), and 3 min post-intubation (T8).

The secondary outcomes included the occurrence of adverse reactions during the induction period, such as respiratory depression, injection pain, hypotension, allergies, bradycardia, muscle tremors, postoperative nausea and vomiting (PONV).

### Adverse effects and management

According to the standard for the phase III trial of ciprofol^14^, adverse reactions are defined as follows: (1) Respiratory depression: SpO_2_ < 90% for > 30 s or respiratory rate < 8 bpm; (2) Bradycardia: HR < 45 bpm or ≥ 20% decrease from baseline value, lasting > 30 s; (3) Tachycardia: HR > 100 bpm or ≥ 20% increase from baseline value, lasting > 30 s; (4) Hypotension: ≥30% decrease in MAP from baseline, lasting > 1 min; (5) Hypertension: ≥30% rise in MAP from baseline value, lasting > 1 min; (6) Injection pain: pain reported in response to questioning and accompanied by a behavioural response, or spontaneous report of pain without questioning; (7) Muscle tremor: tremor in the facial or limb muscle bundles.

During the induction process, if bradycardia occurs, atropine 0.3 mg will be administered intravenously; for tachycardia, adequate sedation is provided, and an appropriate amount of β-blockers is administered intravenously if necessary; and if hypotension occurs, 1 mg of methoxamine or 3 mg of ephedrine is administered intravenously; in the case of hypertension, adequate sedation is provided, and an appropriate amount of urapidil is administered intravenously if necessary.

### Statistical analysis

IBM SPSS Statistics version 26.0 (IBM Corp., Armonk, NY, USA) was used for statistical analysis. *P* < 0.05 was considered statistically significant.

Continuous numerical variables, such as heart rate and blood pressure, were reported as mean ± standard deviation (SD) when normally distributed and analyzed using the independent t-test. For non-normally distributed data, the median [interquartile range (IQR)] was reported, and the Mann–Whitney U-test was used for analysis. Categorical variables, including the incidence of adverse reactions, were expressed as percentages (%) and analyzed using Fisher’s exact test.

Haemodynamic indices at different time points were analyzed using Repeated Measures ANOVA to assess changes over time. The Probit method was used to calculate ED_50_ and ED_95_ and their corresponding 95% confidence intervals (CI) for ciprofol and propofol. Figures were generated using GraphPad Prism 9.5.1 version (GraphPad Software LLC, San Diego, CA, USA).

## Results

### Characteristics of patients

**Fig. 1:**
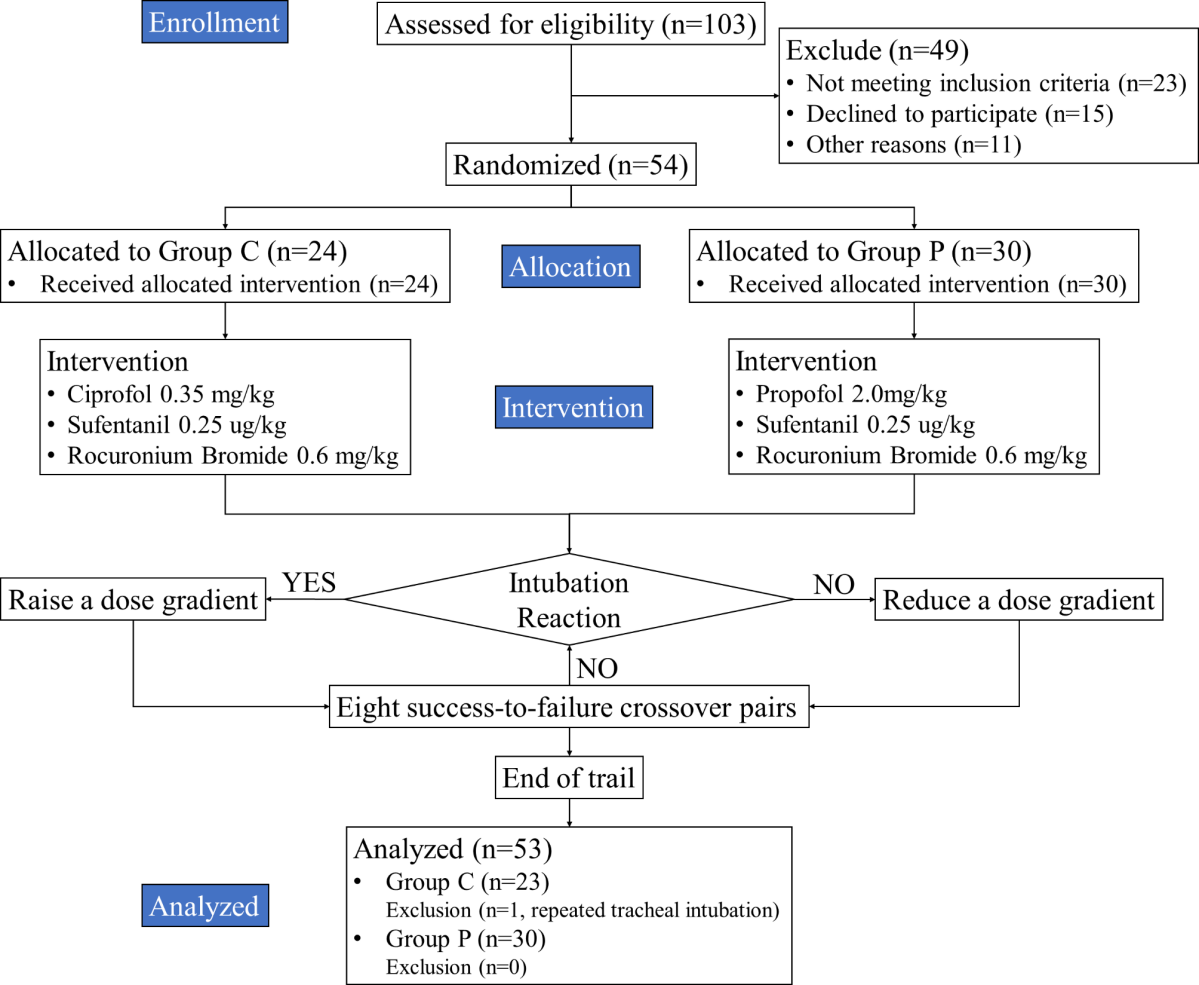
Flowchart of study participant enrollment.

A total of 54 patients were enrolled in this trial, with 24 in group C and 30 in group P. Group C had one exclusion due to repeated tracheal intubation, resulting in a total of 23 patients who completed the study. All patients in group P successfully completed the trial (Fig. 1). No significant differences were found in gender, age, BMI, ASA classification, and Mallampati classifications between groups (*p* > 0.05, Table 2).

**Table 2:** General comparison between Group C and Group P all data are presented as mean ± standard deviation (SD) or n (%).

Group	Group C (*N* = 23)	Group P (*N* = 30)	*p* value
Sex			
Male	9(39.1)	8(26.7)	0.335
Female	14(60.9)	22(73.3)	
Age (years)	47.0 ± 12.3	43.3 ± 10.7	0.596
BMI (kg/m2)	23.2 ± 2.4	22.5 ± 2.5	0.578
ASA class			
Class I	12(52.2)	14(46.7)	0.691
Class II	11(47.8)	16(53.3)	
Modified Mallampati score			
Class I	13(56.5)	16(53.3)	0.817
Class II	10(43.5)	14(46.7)	

ASA, American Society of Anesthesiologists; BMI, body Mass Index.

### Comparison of dose-response and hemodynamic parameters

#### Comparison of dose-response

**Fig. 2:**
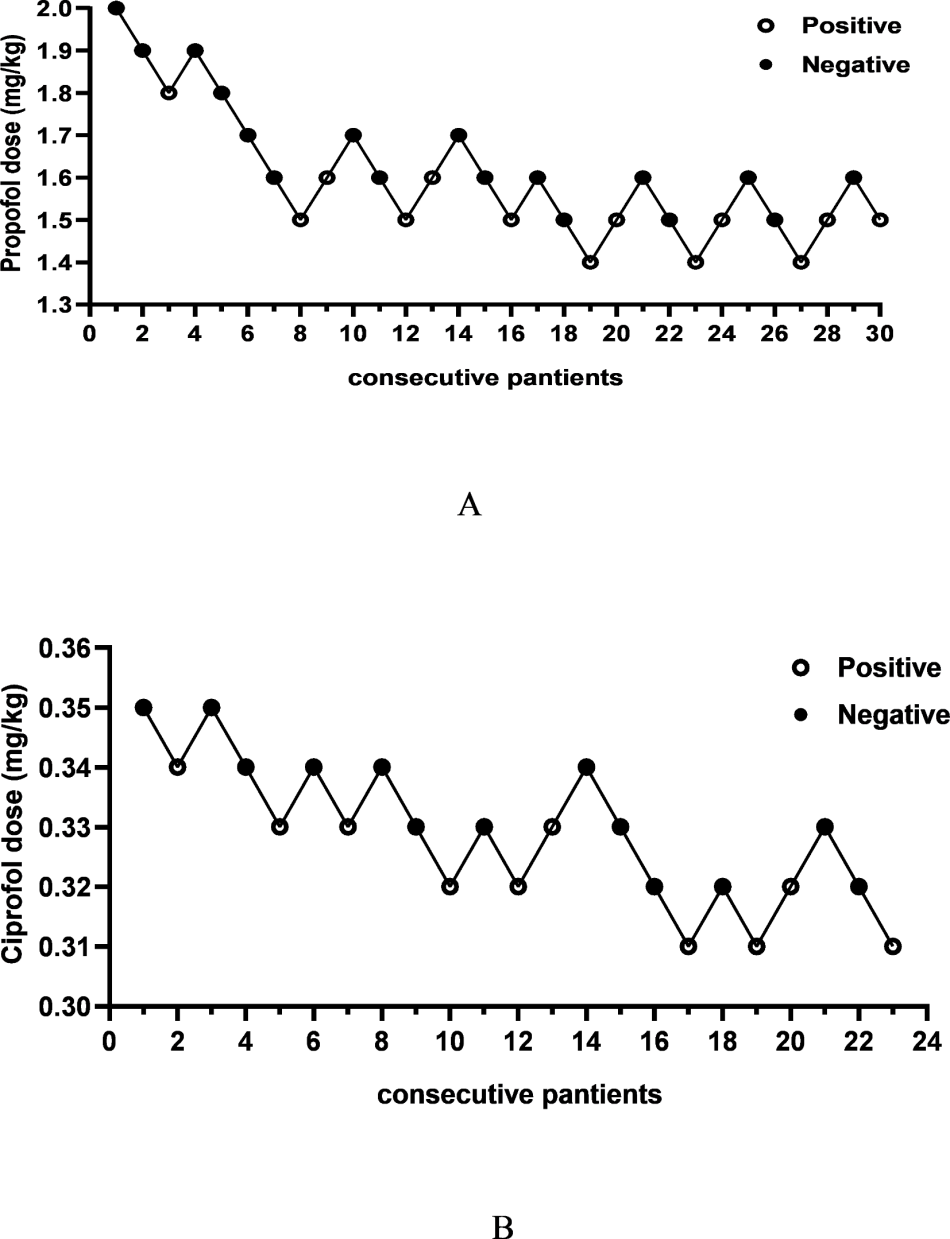
The sequential plots of ciprofol and propofol. Panel A represents the propofol group, panel B represents the ciprofol group. White circles indicate positive tracheal intubation response, while black circles indicate negative tracheal intubation response.

Figure 2 illustrates the positive or negative cardiovascular responses during tracheal intubation in both group C and group P, using the designated dose of the experimental drug for each subject. The x-axis represents the sequence of subject enrollment, while the y-axis represents the dosage of the experimental drug. In group C, 10 patients exhibited positive cardiovascular responses during tracheal intubation, whereas in group P, 13 patients demonstrated positive cardiovascular responses during tracheal intubation.

**Fig. 3:**
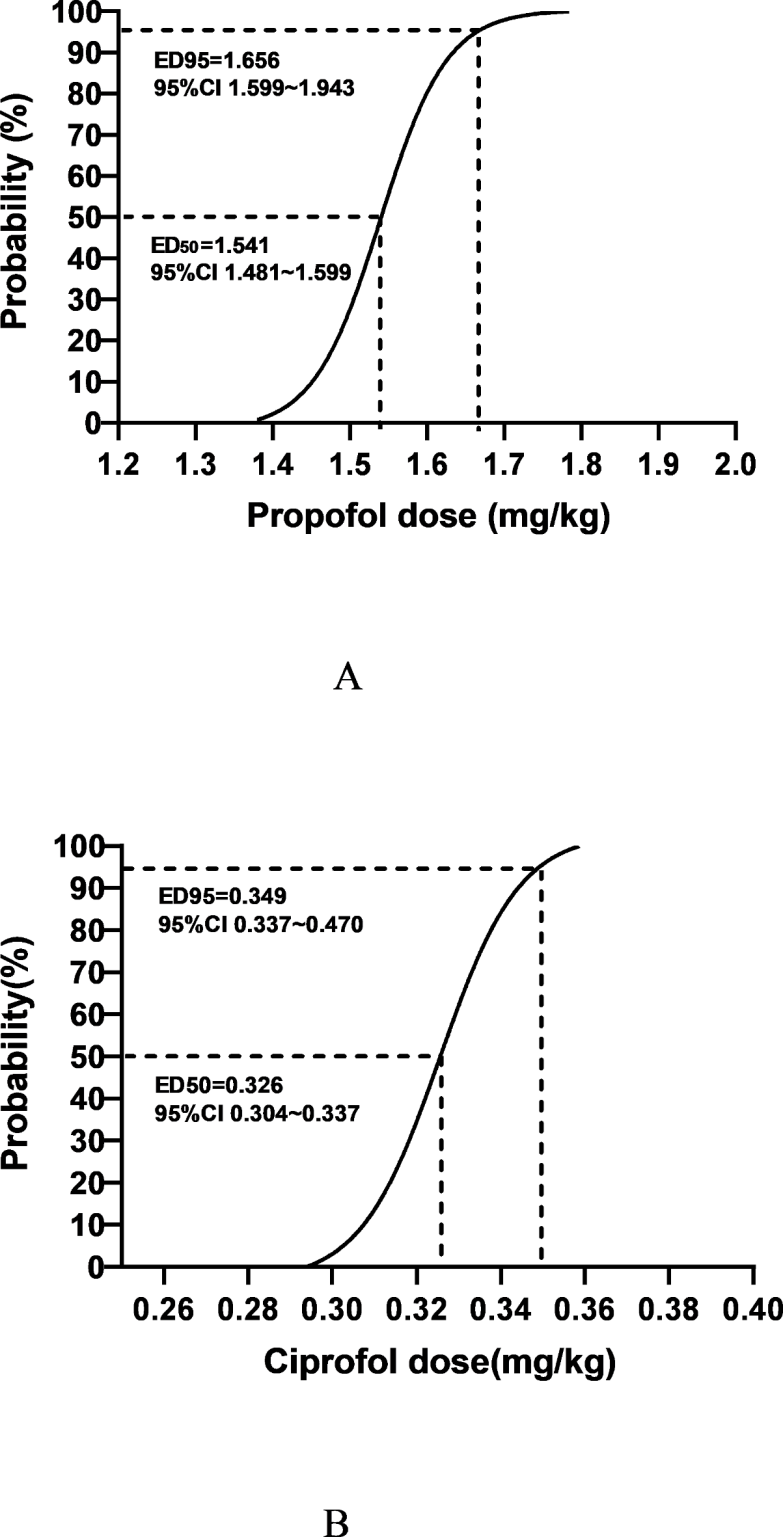
Dose-response curves of propofol and ciprofol. Panel A shows the dose-response curve of propofol, with ED50 and ED95 for inhibiting cardiovascular responses to tracheal intubation being 1.541 mg/kg (95% CI 1.481–1.599 mg/kg) and 1.656 mg/kg (95% CI 1.599–1.943 mg/kg). Panel B shows the dose-response curve of ciprofol, with ED50 and ED95 for inhibiting cardiovascular responses to tracheal intubation being 0.326 mg/kg (95% CI 0.304–0.337 mg/kg) and 0.349 mg/kg (95% CI 0.337–0.470 mg/kg). CI = confidence interval, ED = effective dose.

The ED_50_ and ED_95_ of ciprofol, calculated using the Probit method, for inhibiting cardiovascular responses to tracheal intubation are 0.326 mg/kg (95% CI 0.304 ~ 0.337 mg/kg) and 0.349 mg/kg (95% CI 0.337 ~ 0.470 mg/kg). The ED_50_ and ED_95_ of propofol are 1.541 mg/kg (95% CI 1.481 ~ 1.599 mg/kg) and 1.656 mg/kg (95% CI 1.599 ~ 1.943 mg/kg). The dose-response curves are illustrated in Fig. 3.

#### Comparison of hemodynamic variables

**Fig. 4:**
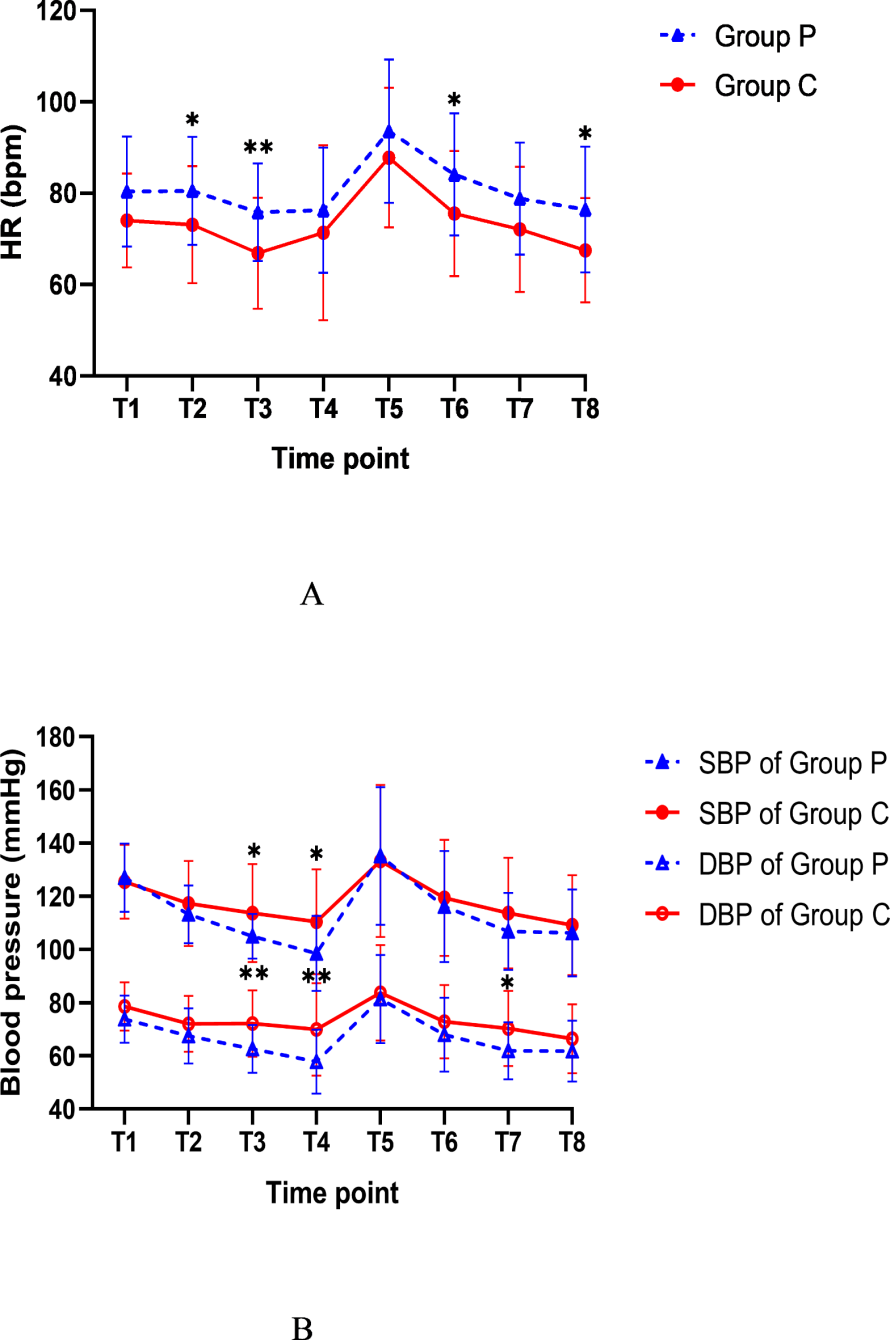
Comparison of BP and HR during induction in Group C and Group P. T1: the average values of three measurements after entering the room as baseline, T2: after intravenous administration of study drug, T3: after complete injection of all drugs, T4: immediately before intubation, immediately after intubation, T5: 1 min after intubation, T6: 2 min after intubation, and T7: 3 min after intubation. * Compared to Group P, Group C showed a *p* ≤ 0.05 for blood pressure or heart rate at the corresponding time point. ** Group C showed a *p* ≤ 0.01 for blood pressure or heart rate at the corresponding time point when compared to Group P.

After intravenous administration of the experimental drug to two groups of patients, there was a significant decrease in blood pressure, which returned to baseline levels 1 min after intubation. In group C, systolic blood pressure (SBP) was significantly lower at T3, T4, T7, and T8 compared to the baseline, while diastolic blood pressure (DBP) was significantly lower at T2, T7, and T8 (*p* < 0.05). In group P, both SBP and DBP were significantly lower before and after intubation compared to the baseline (*p* < 0.05). The heart rate (HR) in group C was significantly lower at T3 compared to the baseline (*p* < 0.05). There were significant differences between group C and group P in terms of DBP and HR (*p* = 0.021 and *p* = 0.016), but no significant difference in SBP (Fig. 4). Post-administration of the drug, HR in group C was lower than in group P (*p* = 0.035); after the administration of all drugs, HR in group C remained significantly lower than in group P (*p* = 0.007), however, both systolic and diastolic pressures in group C were significantly higher than in group P at that time (*p* = 0.025 and *p* = 0.002). At 1 min and 3 min after intubation, HR in group P was higher than in group C (*p* = 0.026 and *p* = 0.016).

#### Adverse reactions

Common adverse reactions in two groups included injection pain and hypotension (Table 3). Group C experienced significantly less injection pain than group P (*p* = 0.001). However, there were no significant differences in the incidence of adverse reactions, such as respiratory depression, hypotension, allergies, muscle tremors, bradycardia, postoperative nausea, and vomiting, between the two groups (*p* > 0.05).

**Table 3:** Comparison of incidence rates of adverse reactions between the two groups.

Adverse reactions	Group C (*N* = 23)	Group P (*N* = 30)	*p* value
Respiratory depression	1(4.3%)	2(6.7%)	1.000
Injection pain	1(4.3%)	15(50%)*	0.001
Hypotension	1(4.3%)	6(20%)	0.208
Allergy	0	1(3.3%)	1.000
Muscle tremors	0	1(3.3%)	1.000
Bradycardia	0	0	-
Postoperative nausea and vomiting	0	0	-

All data are presented as n (%) and *p < 0.05.

## Discussion

As medical technology advances and society develops, public expectations of the healthcare experience continue to rise, resulting in the rapid development of comfort medicine. Reducing irritation caused by tracheal intubation has become a focus of clinical research. Currently, the main ways to reduce the intubation response include the application of sedative drugs, opioids^15^, local anaesthetics, vasoactive drugs and some adjunctive medications. Previous studies have demonstrated that propofol can effectively suppress the gag reflex during intubation and reduce the intubation response^16^. Given the structural similarities between ciprofol and propofol, they have many common points in pharmacokinetics and pharmacodynamics^17^, so we speculated that ciprofol might also be used to suppress the cardiovascular response to tracheal intubation. While numerous studies have confirmed the safety of ciprofol for anesthesia induction^18–20^, direct comparisons between equivalent doses of ciprofol and propofol in suppressing intubation responses remain limited. Therefore, the purpose of our study was to determine the ED_50_ and ED_95_ of ciprofol and propofol for suppressing the cardiovascular response to tracheal intubation and to compare the incidence of adverse reactions between the two drugs. Our findings revealed that the ED_50_ values for ciprofol and propofol in inhibiting the cardiovascular response to tracheal intubation were 0.326 mg/kg and 1.541 mg/kg, respectively. Ciprofol demonstrated more stable hemodynamics and a significantly lower incidence of injection pain during induction compared to propofol.

A randomised controlled study by Xue et al.^21^ reported that administering 0.3 µg/kg sufentanil in combination with propofol for anesthesia induction in children effectively eliminated the cardiovascular intubation response. Similarly, a systematic review by Nazir et al.^22^ confirmed this result but noted that 0.3 µg/kg sufentanil also caused hypotension in unstimulated patients. Given that the pharmacokinetics of sufentanil in children differ significantly from those in adults, the optimal dose for anesthesia induction may vary. Choi et al.^24^ found that 0.3 µg/kg sufentanil effectively reduced cardiovascular responses during double-lumen bronchial intubation in adults without causing other adverse effects. In addition, Chen et al.^25^ reported no statistically significant differences in hemodynamic fluctuations between 0.1 µg/kg and 0.3 µg/kg sufentanil for anesthesia induction in adults. Considering the differences between children and adults, the increased stimulation associated with double-lumen bronchus placement compared to single-lumen tracheal intubation, and the goal of minimizing adverse effects, we selected 0.25 µg/kg sufentanil for anesthesia induction in this study. After intravenous injection of sufentanil, the time to reach peak blood concentration was about 3–5 min^26^. Since the most intense stimulus during general anesthesia induction typically occurs 30 to 45 s after tracheal intubation^27^, we performed tracheal intubation 3 min after administering the induction drug.

The findings of our study suggest that the dosage equivalent to 0.326 mg/kg of ciprofol is 1.541 mg/kg for propofol. Moreover, Ciprofol exhibits a relative potency that is approximately 4.73 times higher than propofol. This aligns with effective doses reported in earlier phase I-III studies and provides a more precise estimation. The effective dose of propofol to inhibit laryngeal mask placement was 1.6 mg/kg in combination with 0.3 mg/kg of esketamine in a study by Lin et al.^28^. Although laryngeal mask placement is less irritating than tracheal intubation, it is worth noting that they did not use a muscle relaxant in their study. And they used a smaller dose of esketamine, which may also account for the slightly higher effective dose of propofol derived from their trial versus ours. In another trial, Min et al. also obtained satisfactory intubation conditions using 1.5 mg/kg of propofol for induction in their study^29^.

Our study reveals a slightly higher effective dose of ciprofol compared to the 0.3 mg/kg recommended by Wu et al.^30^, Ding et al.^31^, and Duan et al.^26^. This variation may be due to the different stimulation intensities between fiberoptic bronchoscopy and tracheal intubation, and the use of topical anesthesia in Wu’s study prior to bronchoscope insertion. It is worth noting that although Ding and Duan used ciprofol for induction of general anesthesia during tracheal intubation, their study subjects focused on elderly participants. The influence of age on the pharmacokinetics and pharmacodynamics of anesthetic drugs may heighten the sensitivity of these patients, consequently necessitating lower effective doses^32^. Contrasting these findings, a recent study by Pei et al.^33^. on pediatric tonsillectomy using ciprofol with low-dose rocuronium bromide reported that the optimal induction dose of ciprofol was 0.6 mg/kg, significantly higher than the doses in our study and other recommendations. This discrepancy may be attributed to variations in experimental protocols, outcome measures, and the unique physiological structure of children. The findings above emphasize the need for anesthesiologists to adjust ciprofol dosage appropriately according to patient-specific factors to enhance anesthesia safety across different procedures and patient populations. Furthermore, additional studies reports that ciprofol doses ranging from 0.3 to 0.5 mg/kg are effective for tracheal intubation^34–36^. Our study builds on these findings, providing a more precise dosage recommendation to enhance the safety and efficacy.

Our study suggests that heart rate was consistently higher in the propofol group compared to the ciprofol group at 1 and 3 min post-intubation (*p* < 0.05). This finding implies that ciprofol may offer myocardial protection by reducing heart rate and subsequently decreasing myocardial oxygen consumption. Therefore, ciprofol could be more advantageous during anesthesia induction in patients with ischemic heart disease, such as coronary artery disease. However, there is currently limited research on this topic, and further studies are needed to substantiate these findings. Intriguingly, studies by Hu et al.^37^ and Bian et al.^9^ observed an increase in heart rate in healthy subjects following ciprofol injection, although this effect emerged more than ten minutes post-injection. Pei et al.^33^ also reported a significant increase in heart rate in pediatric patients after ciprofol administration, which persisted throughout the induction period and tracheal intubation. Given that their study was conducted in children, it is unclear whether this result is linked to the robust compensatory capacity of the pediatric cardiovascular system.

Zhong et al.^38^ reported that the incidence of cardiovascular adverse events related to ciprofol, including hypotension, bradycardia, and prolonged QTc interval, was comparable to that of equipotent doses of propofol. Our findings are consistent with those of Zhong et al., and we noted that blood pressure remained more stable in the ciprofol group compared to the propofol group during induction. Deng et al.^39^ also found that ciprofol maintained higher systolic and diastolic blood pressure values, as well as mean arterial pressure area under the curve, indicating a more stable hemodynamic profile compared to propofol. Similarly, Liao et al.^40^ reached comparable conclusions, but attributed the greater hemodynamic fluctuations in the propofol group due to injection pain and coughing induced by propofol.

Injection pain is a common adverse reaction associated with propofol administration, which can amplify patient tension and anxiety, consequently affecting the stability of anesthesia induction. Our research demonstrated a significantly lower incidence of injection pain with ciprofol compared to propofol (*p* = 0.001). In the ciprofol group, only one case (4.3%) reported mild vein injection pain, whereas 15 patients (50%) in the propofol group experienced this discomfort. This substantial difference highlights the pharmacological advantage of ciprofol in reducing injection pain. Possible contributing factors include its unique chemical structure^41–43^, specific emulsion formulation^44^, high potency^45^, and smaller injection volume required compared to propofol^37^.

Despite the promising findings, our study has several limitations that should be considered. First, the single-center design limits the generalizability of our results to other settings or broader populations. Second, the exclusion of patients with ASA class III or IV restricts the applicability of our findings to higher-risk populations, particularly those with significant comorbidities. Furthermore, we did not examine the long-term hemodynamic effects of ciprofol, which could have important clinical implications, especially during prolonged surgical procedures.

## Conclusion

In summary, our study demonstrates that a ciprofol dose of 0.326 mg/kg is equivalent to 1.541 mg/kg of propofol. Ciprofol administration results in more stable hemodynamics and a reduced incidence of adverse events compared to propofol. These findings indicate that ciprofol could serve as a viable alternative to propofol across various clinical settings, potentially offering improved safety and efficacy during anesthesia induction.

## Data Availability

The datasets used and/or analysed during the current study available from the corresponding author on reasonable request.
